# Wearable K Band Sensors for Telemonitoring and Telehealth and Telemedicine Systems

**DOI:** 10.3390/s25185707

**Published:** 2025-09-12

**Authors:** Albert Sabban

**Affiliations:** Department of Electrical Engineering, Braude College of Engineering, Karmi 2161002, Israel; sabban@braude.ac.il; Tel./Fax: +972-4-875-9111

**Keywords:** wearable sensors, antennas, telemonitoring, telemedicine, fractal antennas, K band

## Abstract

Novel K band wearable sensors and antennas for Telemonitoring, Telehealth and Telemedicine Systems, Internet of Things (IoT) systems, and communication sensors are discussed in this paper. Only in a limited number of papers are K band sensors presented. One of the major goals in the evaluation of Telehealth and Telemedicine and wireless communication devices is the development of efficient compact low-cost antennas and sensors. The development of wideband efficient antennas is crucial to the evaluation of wideband and multiband efficient Telemonitoring, Telehealth and Telemedicine wearable devices. The advantage of the printed wearable antenna is that the feed and matching network can be etched on the same substrate as the printed radiating antenna. K band slot antennas and arrays are presented in this paper the sensors are compact, lightweight, efficient, and wideband. The antennas’ design parameters, and comparison between computation and measured electrical performance of the antennas, are presented in this paper. Fractal efficient antennas and sensors were evaluated to maximize the electrical characteristics of the communication and medical devices. This paper presents wideband printed antennas in frequencies from 16 GH to 26 GHz for Telemonitoring, Telehealth and Telemedicine Systems. The bandwidth of the K band fractal slot antennas and arrays ranges from 10% to 40%. The electrical characteristics of the new compact antennas in the vicinity of the patient body were measured and simulated by using electromagnetic simulation techniques. The gain of the new K band fractal antennas and slot arrays presented in this paper ranges from 3 dBi to 7.5 dBi with 90% efficiency.

## 1. Introduction

The K band printed sensors and antennas presented in this paper are compact, lightweight, wideband, and low-cost. Basic theory and design of small printed antennas is discussed in [1,2]. The low efficiency of small antennas is discussed in [1,2,3,4]. Several types of small efficient wideband wearable antennas are presented in [3,4,5,6,7]. Metamaterials and fractal antennas for wireless communication systems were designed, evaluated, and presented in several publications [3,4,5,6,7,8,9,10]. Printed dipoles, FIPA, and loop antennas, printed slots, microstrip antennas, and other compact antennas are employed in radars, Internet of Things (IoT), 5 G, monitoring, and healthcare systems [2,3,4,5,6,7].

### 1.1. Introduction to Wearable Antennas for Telemonitoring and Telemedicine Systems

Wearable sensors for communication and medical systems were discussed in [4,5,6,7]. Advanced technologies such as metamaterial and fractal structures may be used to improve antennas’ performance. The metamaterial structure and properties define the electrical performance of the metamaterial. Periodic metallic posts and periodic split ring resonators (SRRs) may be used to produce materials with required permeability and dielectric constant, as presented in [5,6,7,8,9,10,11,12,13,14,15,16,17,18]. Metamaterial technology may be used to design small, efficient sensors for communication, Telemonitoring, and Telehealth and Telemedicine Systems [6,7,8,9,10,11,12,13,14,15,16,17,18]. In [8], the development of a metamaterial microstrip antenna was presented; the antenna gain and bandwidth are similar to those of patch antennas. In [9], structures with negative dielectric permittivity are presented. A model and setup to simulate and measure the polarity of SRRs structures are discussed in [10]. Only in a small number of papers are K band sensors presented. “An Improved Performance Radar Sensor for K-Band Automotive Radars” is presented in [11]. A 24 GHz SRR element is used to enhance the operating bandwidth and increase the antenna gain. A dual band transmission-line metamaterial antenna with two transmission line arms is presented in [14]. The antenna bandwidth is 3% with 60% efficiency and 2.6 dBi directivity. The antenna gain is around 0.8 dBi. Compact radiators such as printed loops and dipoles, patches, and FIPA antennas suffer from low efficiency [3,4,5,6,7,17,18,19,20,21,22,23,24,25,26,27,28,29]. These antennas are linearly polarized. Compact efficient metamaterial antennas are a crucial part in wearable Telemonitoring and Telehealth and Telemedicine Systems. In several communication and healthcare systems, the polarization of the receiving signal may be horizontal, elliptical, or vertical. In these cases, the antenna should be dual or circular polarized [19]. Compact efficient wearable metamaterials antennas for communication and medical systems are evaluated in [18,19]. In [20], Small Wearable Metamaterials Antennas for Medical System are presented. Measurements of wearable antenna in the vicinity of the human body are presented in [21]. In [30] a patient remote monitoring system in medical centers is described. Wearable Telemedicine and healthcare sensors are used to increase disease curing and prevention. In [31] a wireless body area network is discussed. A secure thermal-energy aware protocol is presented in [32]. Wearable active sensors and antennas for communication, Telemedicine, and medical applications are presented in [33,34,35,36,37,38,39]. Wearable Telemedicine sensors can monitor and check patients’ daily health [35,36]. Online evaluations of continuously measured medical data of a substantial number of patients can provide low-cost medical treatment. The sensors and antennas presented in this paper may employ in IoT, Internet of Things, and Telemedicine devices. IoT technology is presented in [40]. In [41], Health Monitoring Systems and Wearable Medical Sensors are presented. 

The Importance of Wearables Telemedicine, and Healthcare Sensors in Every Day Life [4,5,6,7,8]

- Wearable sensors and antennas are applied to monitor personal Telemedicine devices to assist elderly, asthmatic, diabetic, and epileptic patients.
- Wearable sensors and antennas may be applied to monitor healthcare activities such as patient health monitoring, patient treatment and care, and personal health monitoring.
- Wearable sensors and antennas may be applied to operate IoT, Telemedicine, and Telehealth devices.

**Figure 1:**
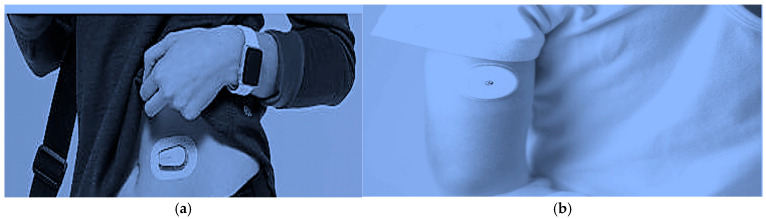
(**a**) Wearable monitoring IoT device. (**b**) Wearable monitoring device using NFC technology.

Advanced antenna design technologies, such as metamaterials and fractal, were used to design efficient ‘wideband antenna and sensors’ [15,16,17,18,19,20,40,41,42,43,44,45,46,47]. Novel advanced technologies such as IoT, artificial intelligence (AI), robotics, and 3D printing are generating new daily life routines, shaping how people buy, exchange, and receive medical treatment, as well as use Telemedicine devices and Telehealth devices. These technologies enhance machine and computer automation, reduce power consumption, and help to create a green-friendly user environment. The Internet of Things (IoT) enables hospitals to monitor, manage, and automate their operations more efficiently and with more control [40]. IoT devices may be a part of green electronic and computing systems. Moreover, wearable IoT devices may make a huge contribution to Telehealth monitoring systems in hospitals and medical centers. Fractal and metamaterial wideband efficient sensors and antennas are an important part of wearable Telemedicine and IoT devices [22,40,41,42,43,44,45,46,47]. Passive and active compact wearable sensors and antennas for medical and IoT applications are discussed in [26,48,49,50,51,52,53]. Wearable medical devices may monitor and evaluate a patient’s daily health [53,54,55,56,57,58,59]. “Monitoring Patient Vital Signs Based on IoT-Based Blockchain Integrity Management Platforms in Smart Hospitals” is presented in [54]. In [59], “Meta-Fractal Wearable Sensors and Antennas for Medical, Communication, and IoT Applications” is presented. The sensors and antennas developed and presented in this paper were evaluated using electromagnetic software [60]. There is a good match between the calculated and measured results on the human body presented in this paper. For electrical characteristics of human body tissues up to 20 GHz are listed, see [61]. The antennas presented in this paper can be used for various data transmission standards such as Bluetooth, Wi-Fi, 5G, and 6G. In [55] a survey about the advanced solutions and technologies that can help IoT-enabled smart grids and blockchain devices is presented. For wearable sensors and antennas can be employed in IoT, smart grid and Telemedicine applications, see [4,5,6,18,19,20,21,62]. In [18,20,53,62], measurement setups and the measured results of sensors and antennas in the vicinity of the user’s body are discussed and presented. A wearable monitoring IoT device is presented in [62]; see Figure 1a. Sensors automatically measure medical data and transmit it to a smartphone. Figure 1b presents a wearable monitoring sensor that provides remote measurement of medical data using NFC technology and a mobile phone or NFC reader. The sensor transmits data at 13.56 MHz

### 1.2. New K Band Wearable Slot Antennas

K band wearable slot antennas are presented in this paper. Slot antennas are dual to dipole antennas. The polarization of the slot antenna is orthogonal to that of a dipole antenna. Electromagnetic fields of a slot antenna may be evaluated by using an equivalent magnetic current. In several wearable systems, the distance separating the transmitting and receiving antennas is less than 2 D^2^/λ. D is the largest dimension of the radiator. In these applications, the amplitude of the electromagnetic field close to the antenna may be quite powerful, but because of rapid fall-off with distance, the antenna do not radiate energy to infinite distances, but instead the radiated power remains trapped in the region near to the antenna. Thus, the near fields only transfer energy to close distances from the receivers. The receiving and transmitting antennas are magnetically coupled. Change in current flow through one wire induces a voltage across the ends of the other wire through electromagnetic induction. The amount of inductive coupling between two conductors is measured by their mutual inductance. In these applications, we have to refer to the near field and not to the far field radiation. In these cases, there is an advantage in using slot antennas. Wideband width may be achieved by using printed slot antennas. In [63], two E-shaped slots are used as RFID sensors for medical applications at 2.5 GHz. Table 1 presents a comparison of the electrical features of several types of wearable antennas and the antennas presented in this paper. Results presented in Table 1 highlight the advantages of the antennas presented in this paper. The slot antennas operate in K bands at 16 GHz to 26 GHz. The new slot antennas are compact and efficient and may be employed in wearable medical applications.

## 2. Wideband K Band T-Shape Slot Antennas for Telemonitoring and Telemedicine Systems

**Figure 2:**
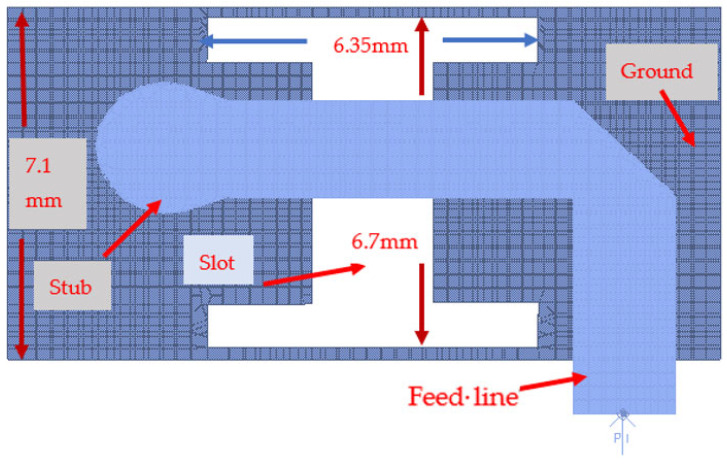
Wideband slot antenna for Telehealth and Telemedicine Systems.

**Figure 3:**
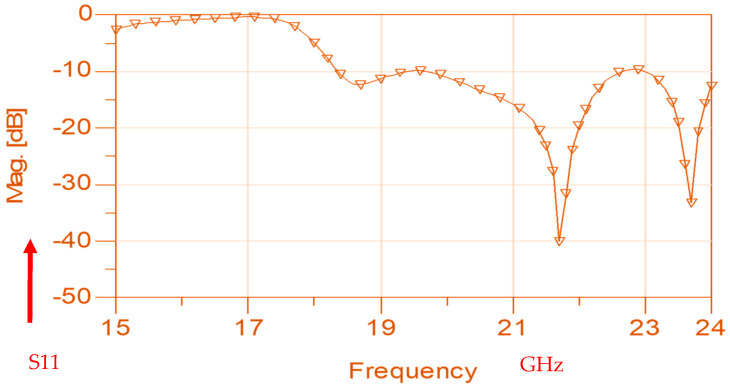
S11 of the wideband slot antenna for Telehealth and Telemedicine Systems.

**Figure 4:**
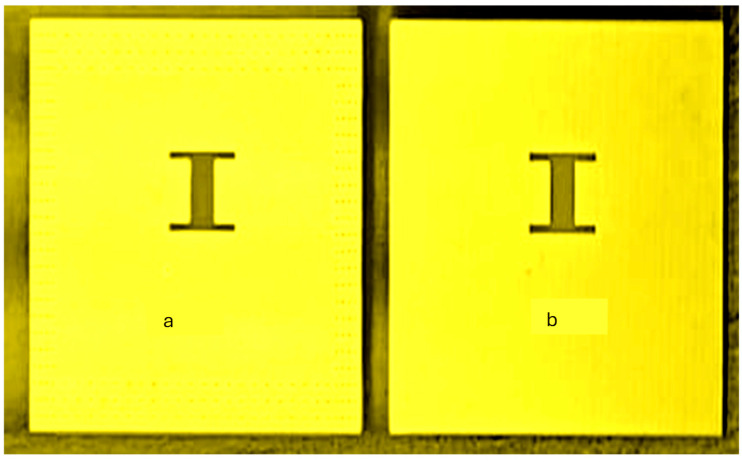
Fabricated wideband slot antenna. (**a**) With via holes. (**b**) Without via holes.

**Figure 5:**
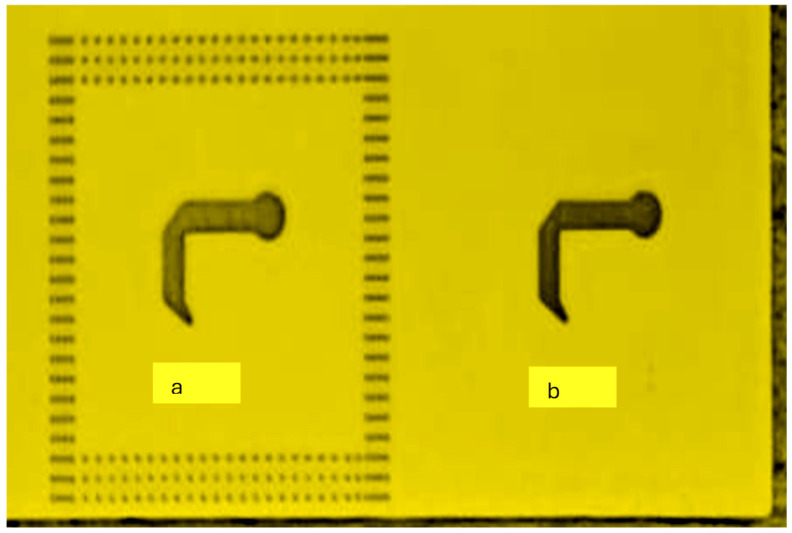
Fabricated feedline of the wideband slot antenna (**a**). With via holes (**b**). Without via holes.

**Figure 6:**
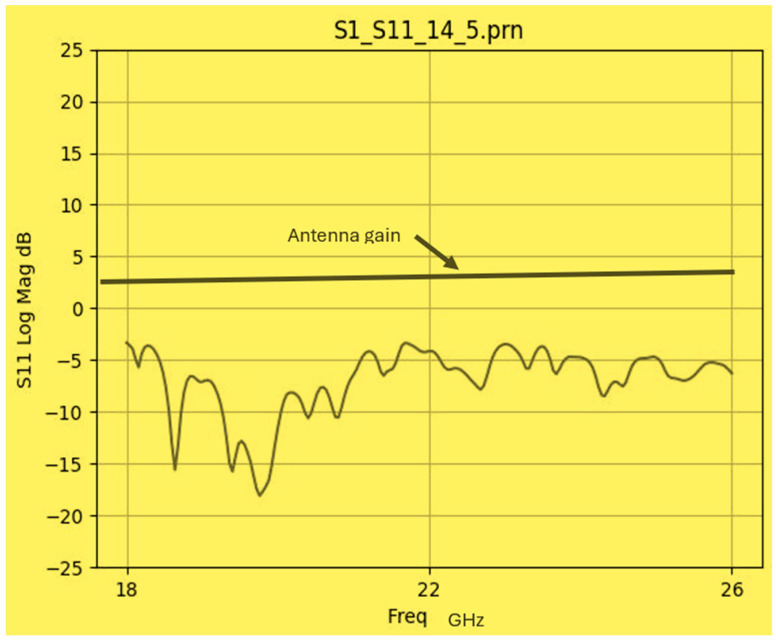
Measured S11 of the wideband slot antenna without via holes.

**Figure 7:**
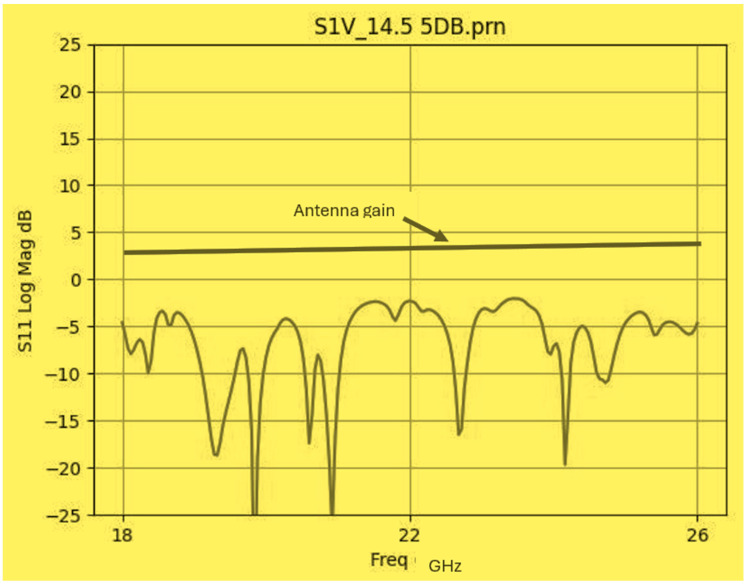
Measured S11 of the wideband slot antenna with via holes.

The wideband K band slot antenna is printed on a dielectric substrate with 3.4 dielectric constant and 0.8 mm thickness. The feed line and the matching network is etched on the same dielectric substrate as the slot antenna. The thickness of the antenna and the feed network, two layers, is 1.6 mm. The antenna layout is shown on Figure 2. S11 of the wideband slot antenna for Telehealth and Telemedicine Systems is shown in Figure 3. The T-Shape slot V.S.W.R is better than 2:1 from 18.5 GHz to 24 GHz. The slot antenna bandwidth is around 26%. The slot antenna beamwidth is around 76° and 3 dBi directivity. The antenna gain is around 3 dBi. The fabricated wideband slot antenna photo with via holes is shown in Figure 4a. The fabricated wideband slot antenna photo without holes is shown on Figure 4b. The fabricated feedline of the wideband slot antenna photo with via holes is shown in Figure 5a. The fabricated feedline of the wideband slot antenna photo without via holes is shown in Figure 5b. The measured S11 of the wideband slot antenna without via holes is shown in Figure 6. The measured S11 of the wideband slot antenna with via holes is shown in Figure 7. In 95% of the frequency range from 18 GHz to 26 GHz the slot antenna V.S.W.R is better than 3:1. The slot antenna without via has better S11 measured results because the antenna matching network of the antenna is optimized to the impedance of the antenna without via holes.

**Figure 8:**
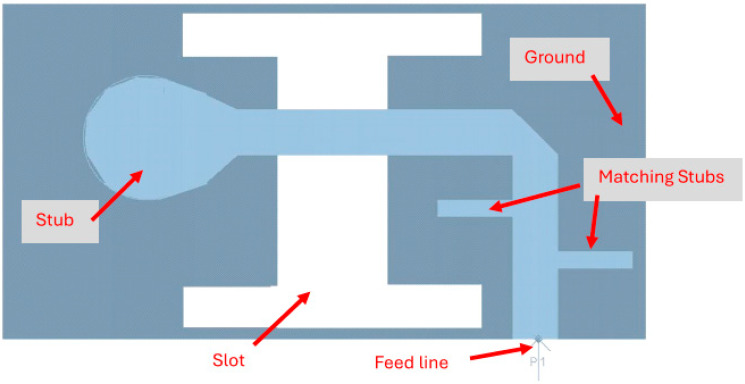
K band Wideband T-Shape slot antenna with matching stubs for Telehealth and Telemedicine Systems.

**Figure 9:**
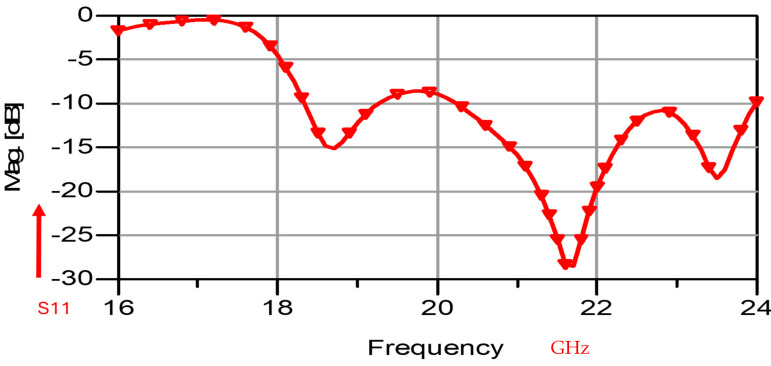
S11 of the wideband modified slot antenna for Telehealth and Telemedicine Systems.

By optimizing the matching network, the electrical performance of the antenna was improved as presented in Figure 8. The T-Shape slot, shown in Figure 8, V.S.W.R is better than 3:1 from 18.0 GHz to 24.5 GHz, as presented in Figure 9. The slot antenna directivity and gain were improved by 2 dB to 3 dB. The antennas and sensors may be attached to the patient skin. The antenna resonant frequency may be shifted by 1%. The skin dielectric constant at 18 GHz is around 30. However, this fact does not affect the sensor electrical specifications due to the wide bandwidth of the sensor around 25%.

The antenna thickness affects the antenna bandwidth. The antenna thickness and dimensions were optimized to achieve the wider bandwidth. Variations to fabrication tolerances are negligible since the antennas have a wideband. Moreover, the antennas are manufacture by using a very accurate printing technology.

## 3. Wideband Fractal K Band T-Shape Slot Antennas for Telemonitoring and Telemedicine Systems

**Figure 10:**
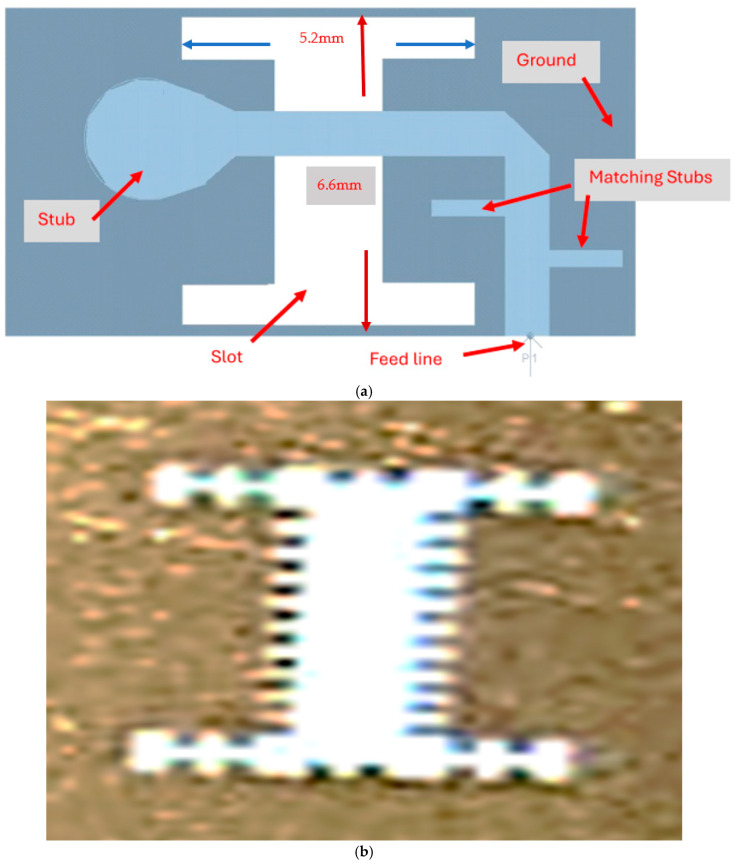
(**a**) Wideband fractal slot antenna for Telehealth Systems (**b**) Fabricated fractal slot antenna.

**Figure 11:**
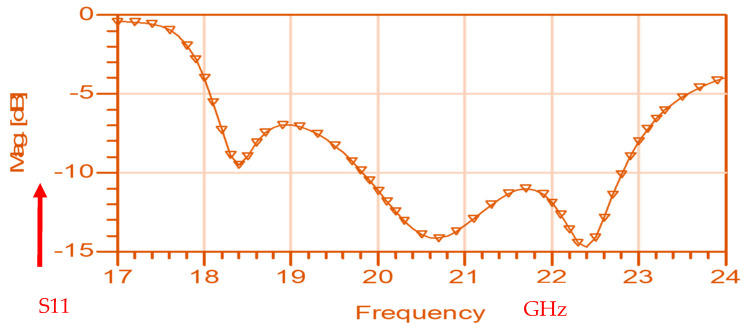
S11 of the fractal wideband slot antenna for Telehealth and Telemedicine Systems.

**Figure 12:**
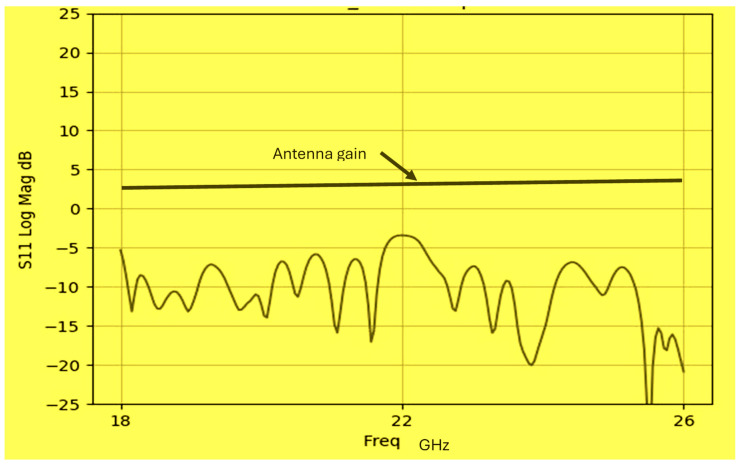
Measured S11 of the fractal wideband slot antenna for Telehealth and Telemedicine Systems.

**Figure 13:**
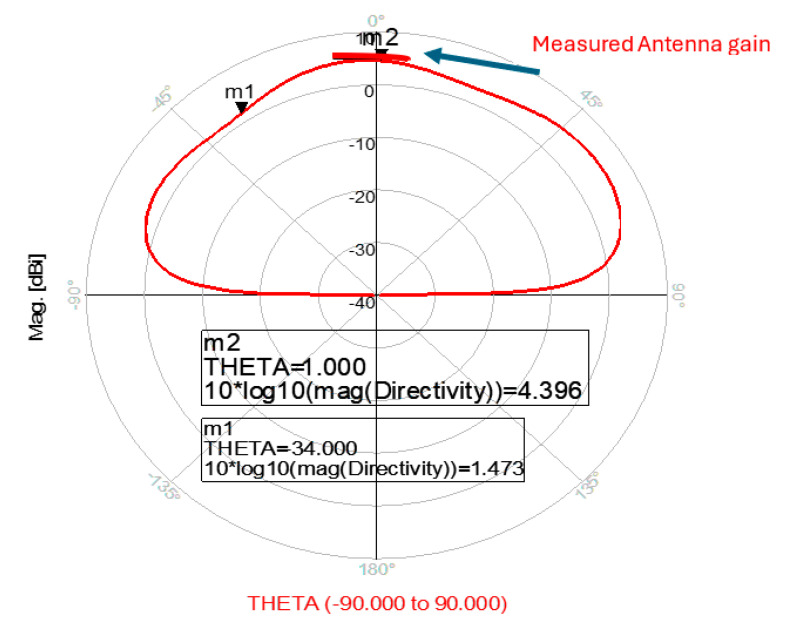
Radiation pattern and measured gain of the wideband fractal slot antenna for Telehealth Systems.

The wideband fractal K band slot antenna is printed on a dielectric substrate with 3.4 dielectric constant and 0.4 mm thickness. However, the layers’ thickness were varied between 0.4 mm and 1.2 mm to optimize the antenna electrical performance. The feed line and the matching network are etched on the same dielectric substrate as the slot antenna with substrate thickness of 0.8 mm. The thickness of the antenna and the feed network, two layers, is 1.2 mm. The antenna layout is shown on Figure 10a and the fabricated fractal slot antenna is shown in Figure 10b. S11 of the wideband slot antenna for medical systems is shown on Figure 11. The fractal T-Shape slot V.S.W.R is better than 3:1 from 18.0 GHz to 23.5 GHz. The fractal T-Shape slot measured S11 results is shown in Figure 12. The measured fractal T-Shape slot V.S.W.R is better than 2:1 in 95% of the bandwidth from 18.0 GHz to 26 GHz. The fractal slot antenna bandwidth is around 26%. The fractal slot antenna beamwidth is around 74° and 4.3 dBi directivity, as presented in Figure 13.

**Figure 14:**
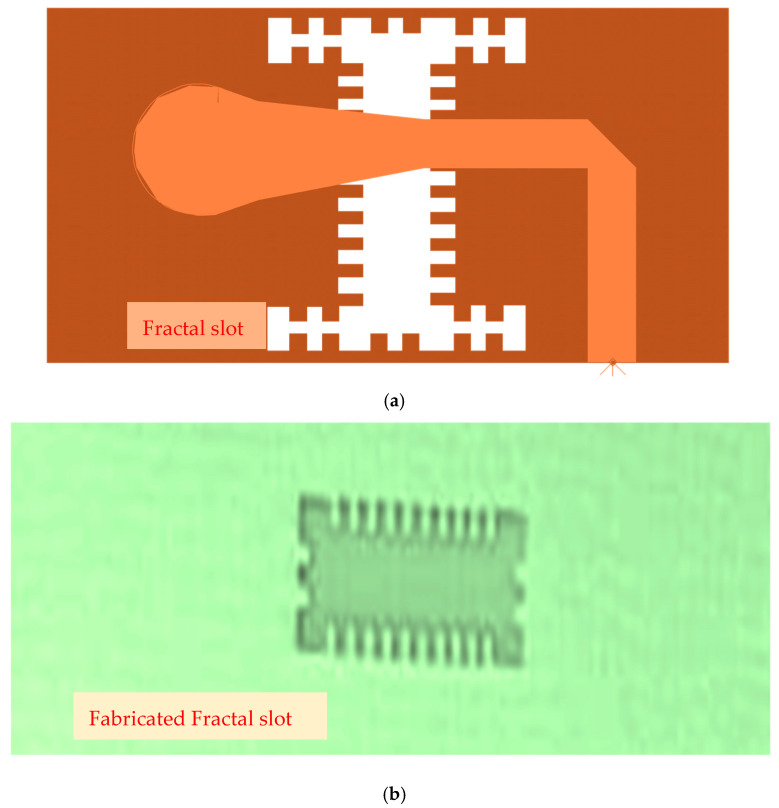
(**a**) Wideband-optimized fractal slot antenna (**b**) Fabricated Wideband fractal slot antenna.

**Figure 15:**
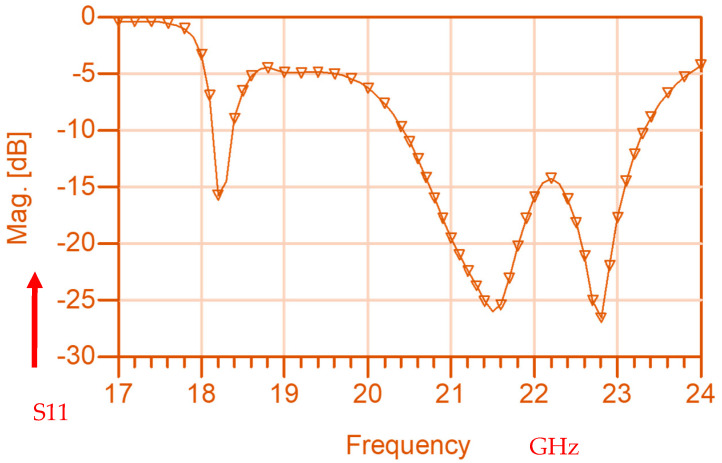
S11 of the fractal wideband slot antenna for Telemedicine Systems.

**Figure 16:**
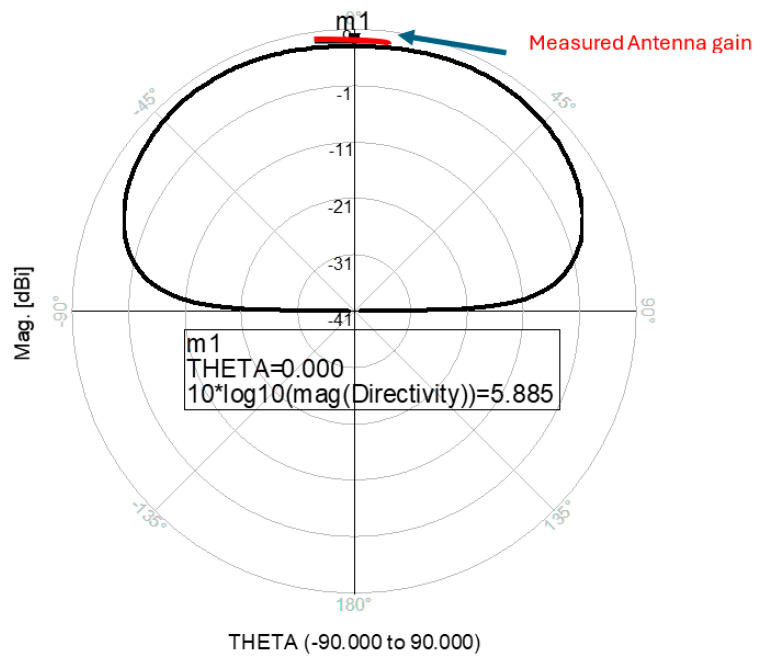
Radiation pattern and measured gain of the wideband modified fractal slot antenna for Telehealth Systems.

**Figure 17:**
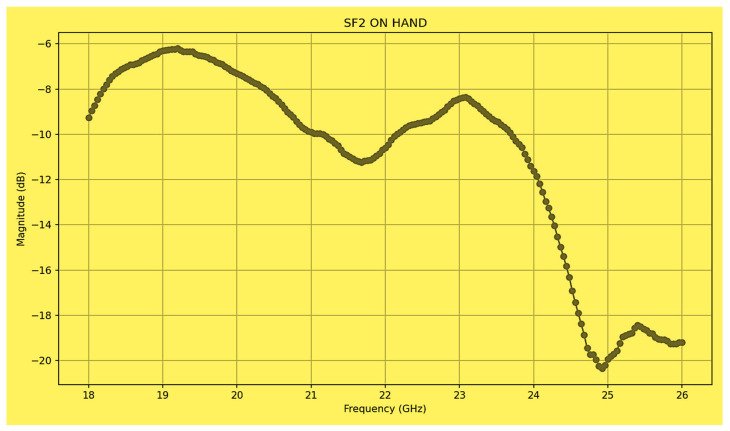
Measured S11 of the fractal wideband slot antenna on the human body.

The antenna gain is around 3 to 4 dBi. By optimizing the matching network and the layers’ thickness, the electrical performance of the antennas may be improved. The thickness of the feed lines layer of the antenna shown in Figure 14a is 0.4 mm. Figure 14b present the fabricated fractal slot antenna. The thickness of the slot layers varied from 0.4 mm to 1.2 mm. The fractal T-Shape slot V.S.W.R is better than 3:1 from 18.0 GHz to 24 GHz, as presented in Figure 15. The fractal slot antenna bandwidth is around 28%. The fractal slot antenna beamwidth is around 76° and 5.8 dBi directivity, as presented in Figure 16. The antenna gain is around 5 to 5.8 dBi. The measured antenna ranges from 5.5 dBi to 5.8 dBi, as shown on Figure 16. The fractal slot antennas in Figure 10a and 14a have the same area. However, by optimizing the matching network and the layers’ thicknesses, the antenna electrical performance was improved. The antenna gain was improved by 1 dB to 2 dB. The measured S11 of the fractal wideband slot antenna on the human body is presented in Figure 17. The fractal antenna VSWR is better than 3:1 in the frequency range from 18 GHz to 26 GHz. The measured fractal T-Shape slot V.S.W.R is better than 2.2:1 in 95% of the bandwidth from 18.0 GHz to 26 GHz on the human body. The fractal slot antenna bandwidth is around 30% for VSWR, better than 3:1.

## 4. Wideband Vertical Fractal K Band Slot Antennas Array for Telemedicine Systems

The wideband fractal K band slot antenna array is printed on a dielectric substrate with 3.4 dielectric constant and 1.2 mm thickness. However, the layer’s thickness were varied between 0.4 mm and 1.2 mm to optimize the antenna electrical performance. The feed line and the matching network are etched on the same dielectric substrate as the slot antenna, with a substrate thickness of 0.8 mm.

**Figure 18:**
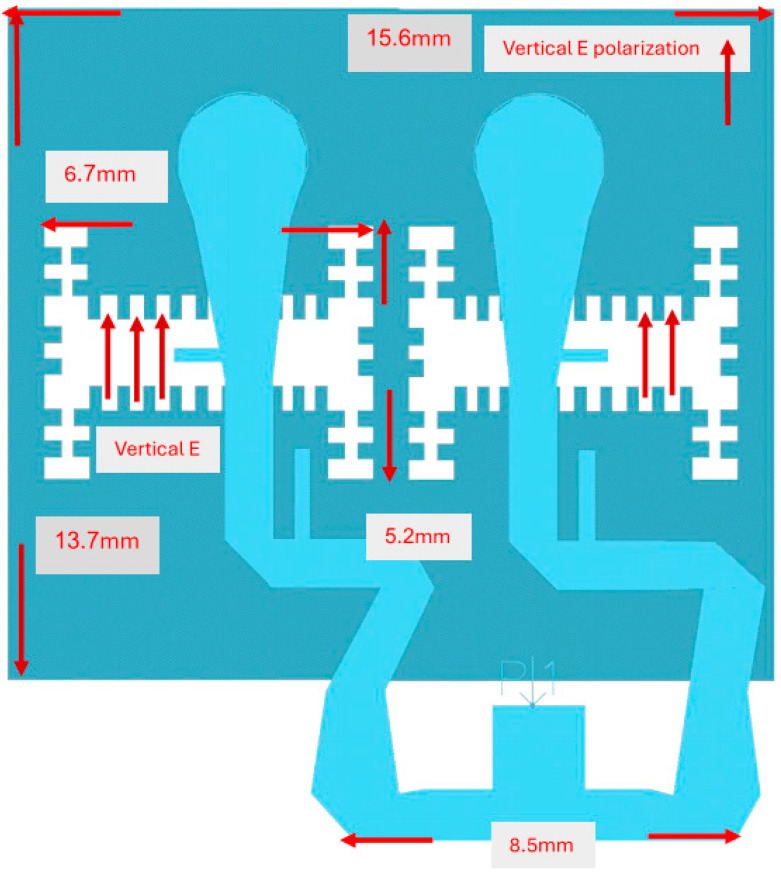
Wideband vertical fractal slot antenna array for Telehealth and Telemedicine Systems.

**Figure 19:**
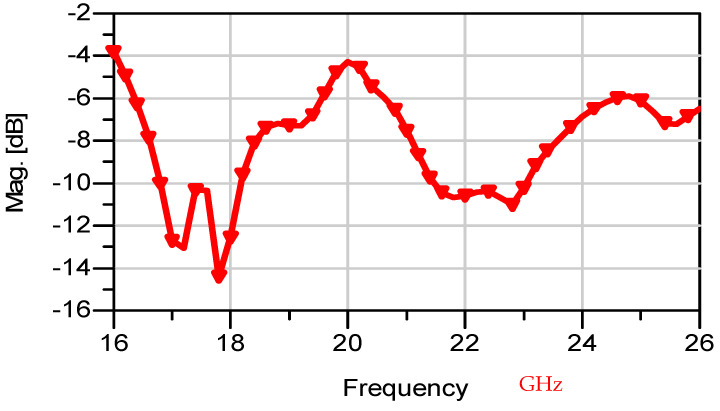
S11 of the vertical fractal wideband slot antenna array for Telemedicine Systems.

**Figure 20:**
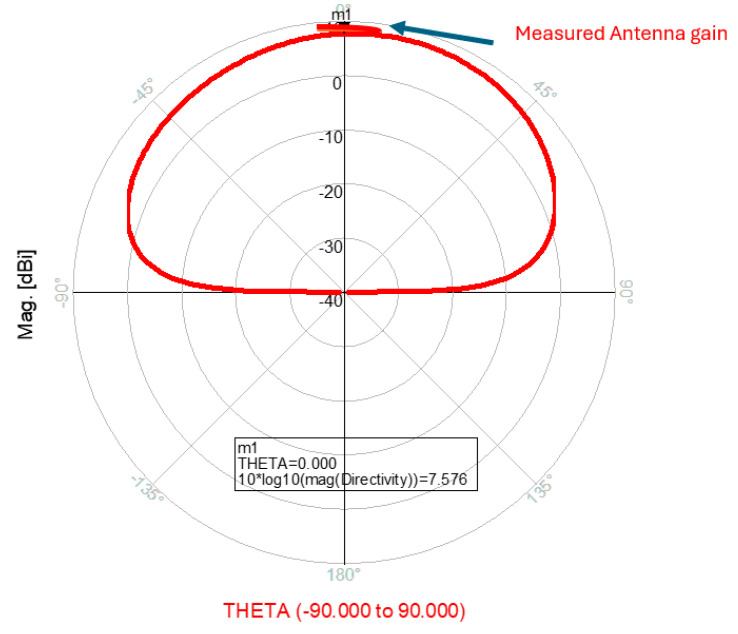
Radiation pattern of the wideband fractal slot antenna array.

**Figure 21:**
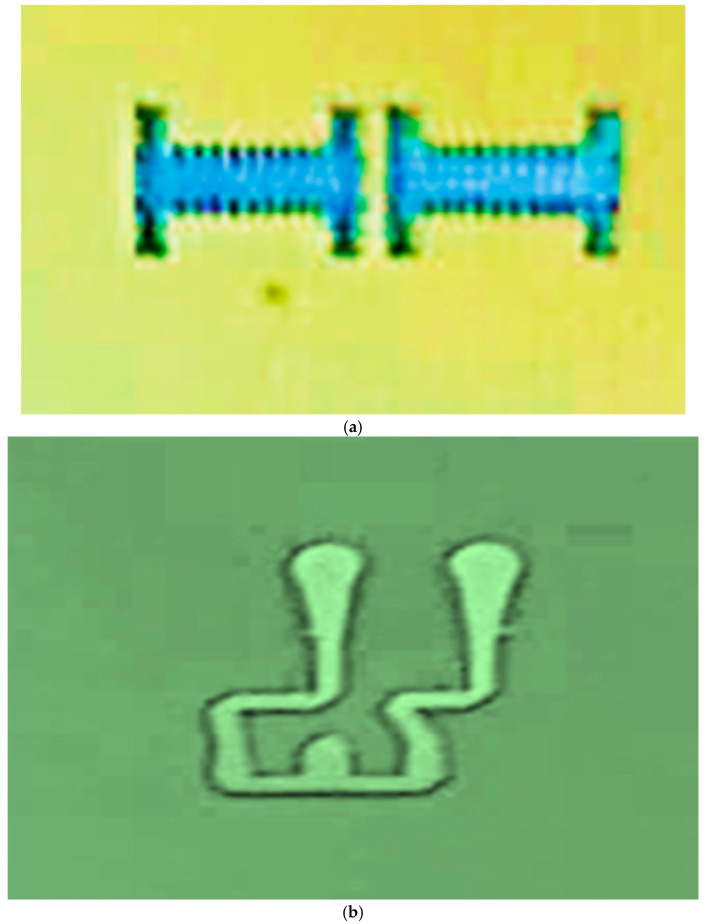
(**a**) Fabricated wideband fractal slot antenna array. (**b**) Feed network of the fabricated fractal slot antenna array.

The thickness of the antenna and the feed network, two layers, is 2 mm. The antenna layout is shown on Figure 18. S11 of the wideband slot antenna for medical systems is shown on Figure 19. The fractal T-Shape slot V.S.W.R is better than 3:1, from 16.4 GHz to 26 GHz. The fractal slot antenna bandwidth is around 45%. The fractal slot antenna array beamwidth is around 70° and 7.5 dBi directivity, as presented in Figure 20. The measured antenna ranges from 7.4 dBi to 7.7 dBi, as shown on Figure 20. The fabricated wideband fractal slot antenna array is presented on Figure 21a and the fabricated feed network of the fractal slot antenna array is presented on Figure 21b.

**Figure 22:**
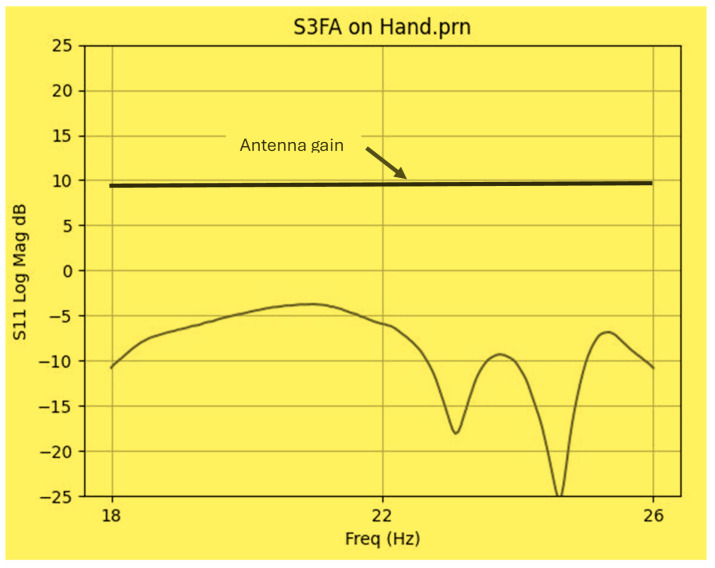
Measured S11 of the vertical fractal wideband slot antenna array on the human body.

The measured fractal T-Shape slot array V.S.W.R is better than 2.2:1 in 90% of the bandwidth from 18.0 GHz to 26 GHz on the human body. The fractal slot antenna bandwidth is around 35% for VSWR, better than 3:1, as presented in Figure 22. The fractal slot antenna array is vertically polarized.

## 5. Wideband K Band Horizontal Polarized Slot Antennas Array for Telemedicine Systems

**Figure 23:**
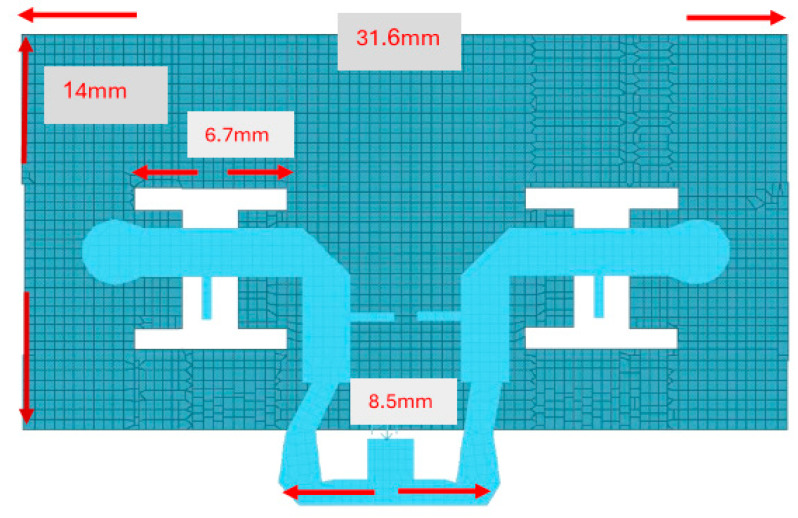
Horizontal wideband slot antenna array for Telehealth and Telemedicine Systems.

**Figure 24:**
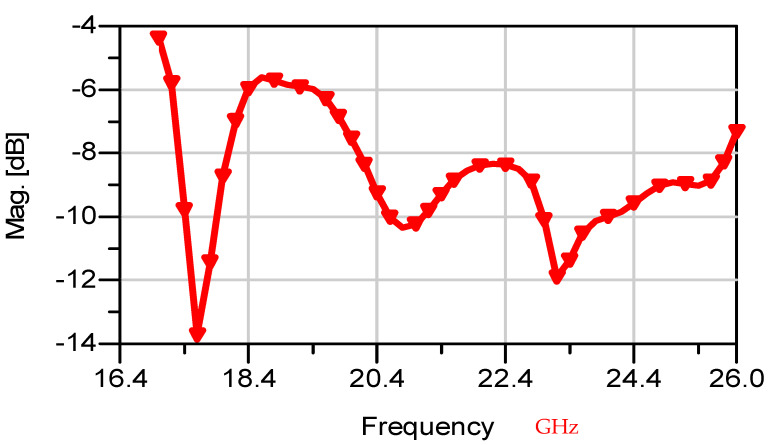
S11 of the wideband slot antenna array for Telemedicine Systems.

**Figure 25:**
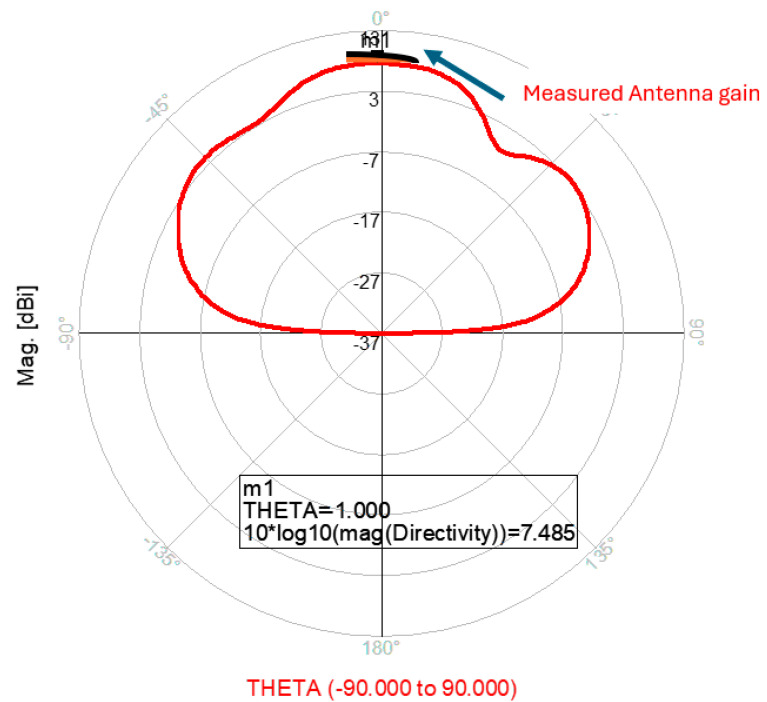
Radiation pattern and antenna gain of the wideband slot antenna array.

**Figure 26:**
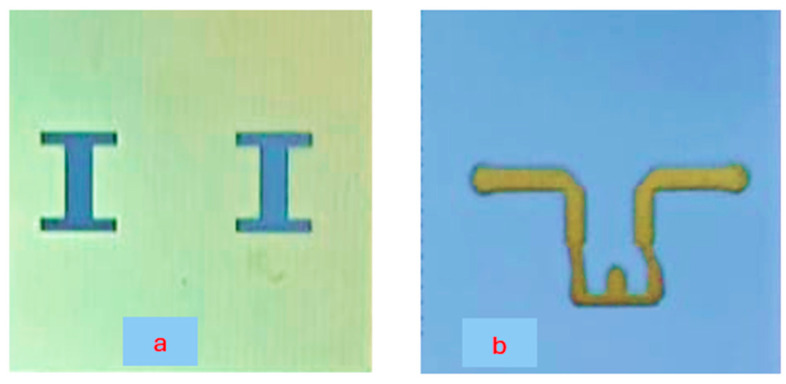
(**a**). Fabricated wideband slot antenna array. (**b**). Feed network of the fabricated slot antenna array.

The wideband K band slot antenna array is printed on a dielectric substrate with 3.4 dielectric constant and 1.2 mm thickness. However, the layers’ thickness were varied between 0.4 mm and 1.2 mm, to optimize the antenna electrical performance. The feed line and the matching network are etched on the same dielectric substrate as the slot antenna with a substrate thickness of 0.8 mm. The thickness of the antenna and the feed network, two layers, is 2 mm. The T-Shape slot dimensions are 6.7 × 6.7 mm as shown on Figure 23. S11 of the wideband slot antenna for medical systems is shown on Figure 24. The fractal T-Shape slot V.S.W.R is better than 3:1, from 16.5 GHz to 26 GHz. The fractal slot antenna bandwidth is around 45%. The fractal slot antenna array beamwidth is around 70° and 7.5 dBi directivity, as presented in Figure 25. The measured antenna ranges from 7.4 dBi to 7.6 dBi, as shown on Figure 25. The fabricated wideband fractal slot antenna array is presented on Figure 26a, and the fabricated feed network of the fractal slot antenna array is presented on Figure 26b.

## 6. Applications of Wideband K Band Sensors for Telemonitoring and Telemedicine Systems

**Figure 27:**
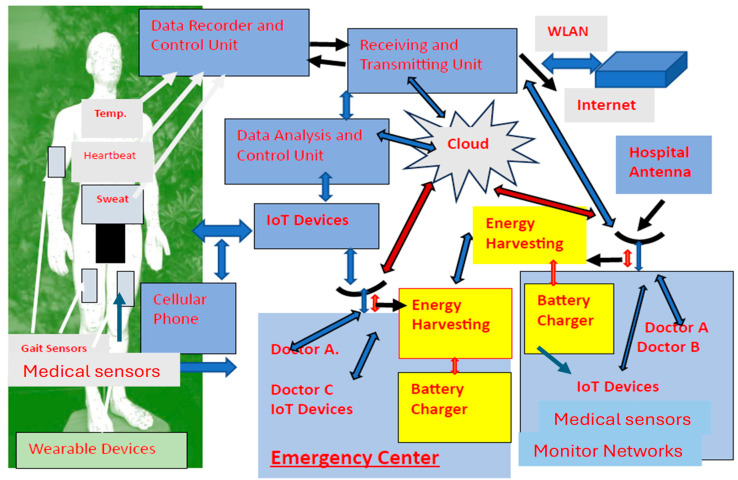
Applications for wearable sensors in healthcare centers, Telemedicine Systems, and IoT devices.

Applications of wearable sensors in healthcare centers, Telemedicine Systems, and IoT devices, where the medical parameters of large numbers of patients are constantly being monitored, is presented in Figure 27. By using wearable medical devices, physicians may rapidly evaluate and diagnose patients. As shown in Figure 27, energy harvesting units may be connected to the Telemedicine and IoT devices to provide green renewable energy.

Wearable devices and sensors are important part of IoT devices and systems. Wearable sensors and antennas can telemonitor medical networks, hospitals’ facilities, and daily procedures such as patient heartbeat, blood pressure, patient temperature, and other parameters. Telemedicine monitoring compact wearable sensors can monitor and track personal data about the user health signs such as heartbeat, patient temperature, sweat, blood pressure, calorie budget and other parameters.

**Figure 28:**
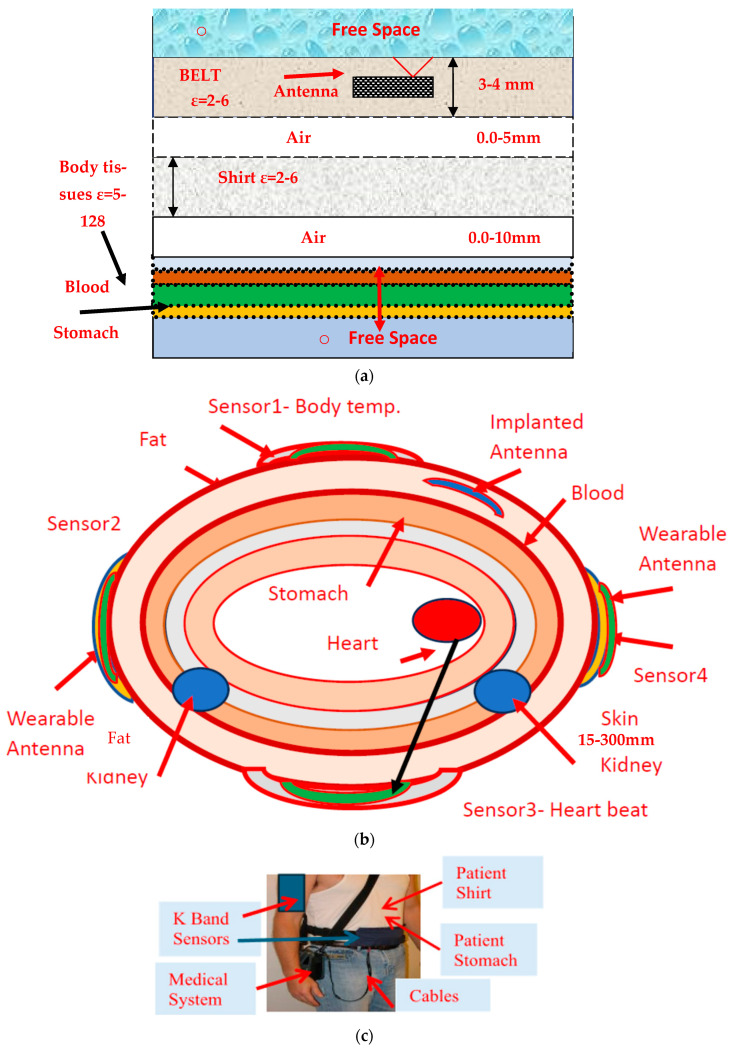
(**a**) Analysis model of wearable antennas. (**b**) Medical wearable sensors on human body. (**c**) K band sensors on human body.

The wearable sensors’ electrical characteristics variation near the human body were simulated by generating a model of the human body and the antenna, as shown in Figure 28a. Medical wearable sensors on the human body are shown in Figure 28b. K band sensors on human body are presented in Figure 28c. K band sensors may be attached to the patient’s hands, stomach, or back. The influence of the human body on the sensors’ performance is simulated by evaluating the antenna reflection coefficient on the user body. The variation in the electrical characteristics of the body tissues affects the electrical performance of the sensors. In Table 2, electrical characteristics of human body tissues up to 20 GHz are listed; see [61]. The sensor resonant frequency is shifted between 2% and 5%, in various locations of the sensor on the patient body. The antenna’s electrical and mechanical features were optimized and tuned to achieve the best sensor electrical and mechanical characteristics.

Table 3 presents simulation and measured results of fractal slot wearable antennas discussed in this paper. As presented in Table 2, there is a good agreement between the computed and measured results. In [18,20,53,62], measurement setups and the measured results of sensors and antennas in the vicinity of the user’s body are discussed and presented.

**Figure 29:**
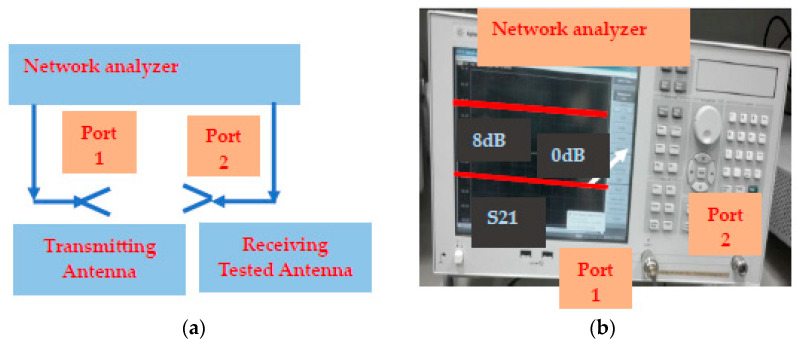
(**a**) Gain measurements. (**b**) Network analyzer S21 measurements.

The antennas and the S_11_ and S_21_ sensors’ parameter measurements were conducted by using a two-port calibrated network analyzer, as shown in Figure 29. The sensor’s gain is obtained by measuring S_21._ The two-port S parameter measurement setup is shown in Figure 29.

The antennas presented in this paper are part of the communication systems that transmit short pulses. The absorption rate, or the SAR in these cases, is low. Moreover, SAR computation is important for transmitting systems. The transmitted power of the sensors presented in this paper is lower than 10 dBm. This power rate was approved by the FDA, the Food and Drug Association for communication devices.

## 7. Conclusions

New K band sensors and antennas for Telemonitoring, Telehealth and Telemedicine Systems, Internet of Things (IoT) systems, and communication sensors are presented and discussed in this paper. Only a few papers on K band wideband efficient sensors are presented. Novel compact wideband K band slot arrays are presented in this paper. The new antennas may be used in commercial IoT and Telemedicine devices.

Wideband compact printed antennas in frequencies from 16 GH to 26 GHz for Telemonitoring and Telehealth and Telemedicine Systems are evaluated in this paper. The size of the fractal slot antennas is 20 × 20 × 2 mm. The bandwidth of the K band fractal slot antennas and arrays ranges from 10% to 40%. The electrical characteristics of the new compact antennas in vicinity to the patient body were measured and simulated by using electromagnetic simulation techniques. The gain of the new K band fractal antennas and slot arrays presented in this paper ranges from 3 dBi to 7.5 dBi with 90% efficiency. There is a good agreement between measured and computed results. The size of the fractal slot antenna array is 30 × 30 × 2 mm. The bandwidth of the fractal slot array is around 45% with 7.8 dBi gain. The efficiency of the fractal slot antennas is around 90%.

In future research, more efficient compact K band antennas will be designed. Metamaterial fractal sensors will be evaluated and manufactured.

## Figures and Tables

**Table 1:** Wearable Antennas Bandwidth, Gain and Efficiency Comparison.

Antenna Type	Freq. GHz	Gain dB	BW %	Effic. %	Beamwidth		Application Smart Devices	Reference
					θE°	θH°		
**Printed dipole**	0.1–12	2–3	5–8	80	90	90	Communication	[6,7,8]
**Patches**	1–40	3–4	1–2	80	90	90	Medical, 5G, IoT	[6,7,8]
**Stacked Fractal**	1–18	7.8	8–10	91	76	78	Medical, 5G, IoT	[6,7,8]
**Meta-Fractal** **Patch**	1–12	8	20	90	76	90	Medical, 5G, IoT, Smart devices	[59]
**Slot**	16–26	2–4	26	90	76	90	Medical, 5G, IoT	Present paper
**Fractal Slot**	16–26	3–5	26	90	76	90	Medical, 5G, IoT	Present paper
**Slot Fractal Array**	16–26	7–8	45	70	70	90	Medical, 5G, IoT	Present paper
**Slot Array**	16–26	7–8	45	70	70	90	Medical, 5G, IoT	Present paper
**Active Antennas**	1–6	13	50	90	74	74	Medical, 5G, IoT	[6,7,8]

**Table 2:** Electrical parameters of human body tissues [22,23,61].

Tissue	Parameter	0.6 GHz	1 GHz	2 GHz	5G Hz	10 GHz	20 GHz
Fat tissues	σ	0.05	0.054	0.1	0.3	0.5	1
	ε	5.00	4.72	4.5	4.3	4.2	4.0
Muscle tissues	σ	0.8	1	1.5	5	11	22
	ε	56	55	53	50	40	25
Blood	σ	1.8	1.9	2	5	15	28
	ε	59	58	56	51	40	25
Skin	σ	0.6	0.7	1	3	9	20
	ε	46	45	43	40	33	23

**Table 3:** Simulation and measured results of fractal slot wearable antennas.

Sensors	Frequency (GHz)	BW %	BW% Measured	Computed Gain dBi	Measured Gain dBi *	Antenna Area (cm)	Efficiency ** %
K band slot Figure 2	18–26	36	36	3	3–4	1.5 × 1.5	86–92
K band slot Figure 4	18–26	32	32	4	4–4.3	2 × 2	88–92
Fractal slot Figure 10	18–26	25	26	4	4–4.5	2 × 2	85–90
K band slot Figure 14	18–26	28	28	5.5	5.5–5.8	2 × 2	86–92
Fractal array Figure 18	16–26	45	45	7.5	7.5–7.8	3 × 3	85–90
Slot array Figure 23	16.4–26	44	44	7.5	7.5–7.8	3 × 3	85–90

* The gain varies from 5.5 to 5.8 dBI in the frequency range from 18 to 26 GHz. The gain presented in Table 3 represents gain variation in the frequency range from 18 to 26 GHz. Moreover, this is an exceedingly small variation in the frequency range from 18 to 26 GHz. The accuracy of gain measurement in the frequency range from 18 to 26 GHz is around ±0.5 dB. ** Efficiency is calculated by electromagnetic software ADS [60]. Efficiency varies from 85% to 92% in the frequency range from 18 to 26 GHz. Losses are due to losses in the antenna feed network and matching losses.

## Data Availability

Data are unavailable due to privacy.
